# Take-off and landing techniques in snowboard halfpipe based on inertial measurement units and kinematics analysis

**DOI:** 10.7717/peerj.21778

**Published:** 2026-09-25

**Authors:** Jinglun Yu, Qi Li, Shengnian Zhang, Quan Zhou

**Affiliations:** 1 School of Exercise and Health Sciences, Xi’an Physical Education University, Xi’an, China; 2 Key Laboratory of Sports Technology Analysis and Skill Assessment of General Administration of Sport, Xi’an Physical Education University, Xi’an, China; 3 Key Laboratory of Exercise and Health Sciences of Ministry of Education, Shanghai University of Sport, Shanghai, China; 4 Foundational Department, Shandong Xiehe University, Jinan, China

**Keywords:** Snowboard halfpipe, Take-off, Landing, IMU, Kinematics

## Abstract

**Background:**

The unique characteristics of snowboard halfpipe projects present a research challenge. Inertial measurement units (IMUs) show promise in addressing the prevailing research problems due to their numerous advantages. This study aims to explore the key technical characteristics of the take-off and landing phases in snowboard halfpipe to analyse the key factors that affect the take-off quality and landing stability of athletes at different sport levels.

**Methods:**

The study used an IMU-based motion capture system combined with high-speed video recording (*via* Xsens and Fastcam Mini WX100 high-speed cameras) to collect kinematic data from halfpipe snowboarders during their movement process.

**Results:**

The elite athletes reached the vertical position at approximately 50% of the normalized take-off phase, compared with approximately 60% in novice athletes. The overall speeds achieved by the elite athletes were greater than those of the novice athletes. The elite group demonstrated a lower flexion range of motion and a stiffer landing pattern than the novice group.

**Conclusion:**

These findings suggest that snowboard halfpipe performance may depend on the coordinated control of take-off timing, snowboard inclination, and landing posture.

## Introduction

Snowboard halfpipe is an Olympic snowboard event and is part of the skill-oriented events that are characterised by difficulty and beauty (Malcata & Hopkins, 2014). The event was listed as an official competition at the 1998 Nagano Winter Olympics. This event requires snowboarders to use a snowboard to glide in a U-shaped halfpipe with a length of 100–200 m, a width of 14–22 m, a depth of 3.0–7.5 m and a slope of 13.0°–18.7° (Li, Guo & Wu, 2017). Athletes use both walls to propel themselves into the air and perform complex tricks as they navigate through the halfpipe, aiming to complete a run judged on difficulty, amplitude, variety and execution (Su & Zhu, 2020; Bao & Wang, 2013). A complete movement phase within the field can be subdivided into in-run, take-off, flight and landing. In addition to the simple style action ‘first hit’, which is used for the first time to search for sensation, the pre-designed high-difficulty multi-axis rotation movements in the air will have a take-off movement in the take-off phase and a landing action in the landing phase before and after to connect (Yan, Liu & Guo, 2009). The quality of this transition technique and the choice of line will have an irreparable influence on the completion quality of the next action (Merz et al., 2025a). Therefore, the take-off and landing phases and their influencing factors warrant detailed examination in snowboard halfpipe.

At present, research on snowboard halfpipe has examined the sport from different perspectives, including technical theoretical analysis, lower limb isokinetic muscle strength and core stability testing (Wang, Liu & Qi, 2014), sport injury investigation (Seleznev et al., 2018; Weinstein, Khodaee & Vanbaak, 2019), muscle characteristics (Sun et al., 2018), velocity-related factors (Yan, Liu & Guo, 2009; Jiang et al., 2012), and fatigue monitoring during training (Turnbull, 2013). More recently, biomechanical and sensor-based studies have investigated snowboard halfpipe and snowboard freestyle in greater detail, including take-off and landing characteristics, in-field biomechanics, Inertial measurement units (IMU)-based event detection, foot-specific acceleration, ground reaction forces, and wearable- or video-based assessment of rotational parameters, as well as the biomechanical determinants of rotational performance (Kersting et al., 2022; Thelen et al., 2024; Thelen et al., 2025; Gorges et al., 2024; Gorges, Thelen & Merz, 2024; Gorges et al., 2025; Merz et al., 2025b; Merz et al., 2026). However, quantitative and biomechanical research on the key technical movements in snowboard halfpipe remains insufficient, particularly regarding the take-off and landing phases under real field conditions. This phenomenon arises because traditional sport biomechanical measurement methods cannot be conducted in environments with complex terrain, low temperature and large range of motion in snowboard halfpipe, posing significant limitations and difficulties in research. In recent years, IMUs have been widely used. These IMUs exhibit the advantages of high precision, small size and strong portability, making them well suited for sport training and performance feedback (Chardonnens et al., 2012; Chardonnens et al., 2014; Bessone, Petrat & Schwirtz, 2019; Gorges et al., 2025). Moreover, IMUs are highly suitable for complex kinematic research and have the potential to address the aforementioned problems. The application of IMUs in snow events, including ski jumping (Yu et al., 2023), cross-country skiing (Fasel et al., 2015) and ski mountaineering (Gellaerts et al., 2018), is becoming increasingly widespread and has recently been extended to snowboard halfpipe (Thelen et al., 2024; Gorges et al., 2024; Gorges, Thelen & Merz, 2024).

Based on the technical structure of snowboard halfpipe, the importance of movement continuity between the take-off and landing phases, and previous biomechanical research on snowboard halfpipe performance, this study focused on take-off and landing as the key technical phases. The research objectives were to explore the movement characteristics of these phases and to analyse the main factors affecting take-off quality and landing stability in athletes of different performance levels. In particular, timing control, snowboard inclination, and posture regulation were selected as the main factors of interest based on these technical and biomechanical considerations. The study also aimed to provide quantitative evidence to support technique improvement and training guidance for athletes and coaches. The research hypothesis is that technical differences between athletes could be identified due to technical proficiency, and that elite athletes would have more reasonable and stable take-off and landing movements compared with novice athletes.

## Materials & Methods

### Study design

#### Equipment layout

The test site was the U-shaped halfpipe of the Jilin Changbai Mountain, with a length of 120 m, a width of 16 m, a slope of 16° and a vertical inclination of 86°. The halfpipe met the requirements of international snow competitions.

In the study design, Fastcam Mini WX100 high-speed camera A was placed on one side of the deck to focus on capturing the first complete movement (in-run, take-off, flight and landing, Fig. 1A). Fastcam Mini WX100 high-speed camera B was placed on the finish area to focus on capturing the movement trace of the halfpipe snowboarders (Fig. 1B). The cameras were set up on a professional fixed tripod.

**Figure 1:**
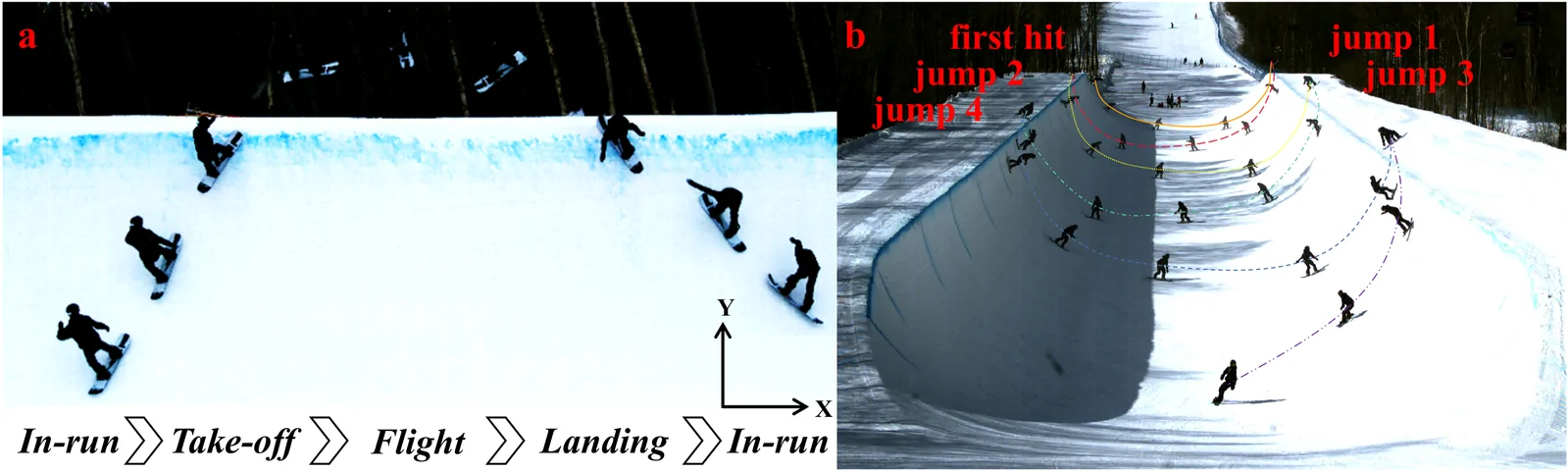
Camera views showing the movement phases and overall movement trace in the snowboard halfpipe.

A red flag was placed on the deck opposite Camera A as a marker in advance. The athletes were instructed to perform the first jump immediately after passing the red flag, so that the take-off location of the first jump was kept as consistent as possible across athletes and could be clearly captured by Camera A. Camera A was used only to record the first jump. Camera B was positioned at the bottom of the U-shaped halfpipe to record the athletes’ overall movement trace and line throughout the run (Fig. 2B). The athletes wore the Xsens suit. The captured videos were stored on a computer hard disk, and the Xsens data were stored offline.

**Figure 2:**
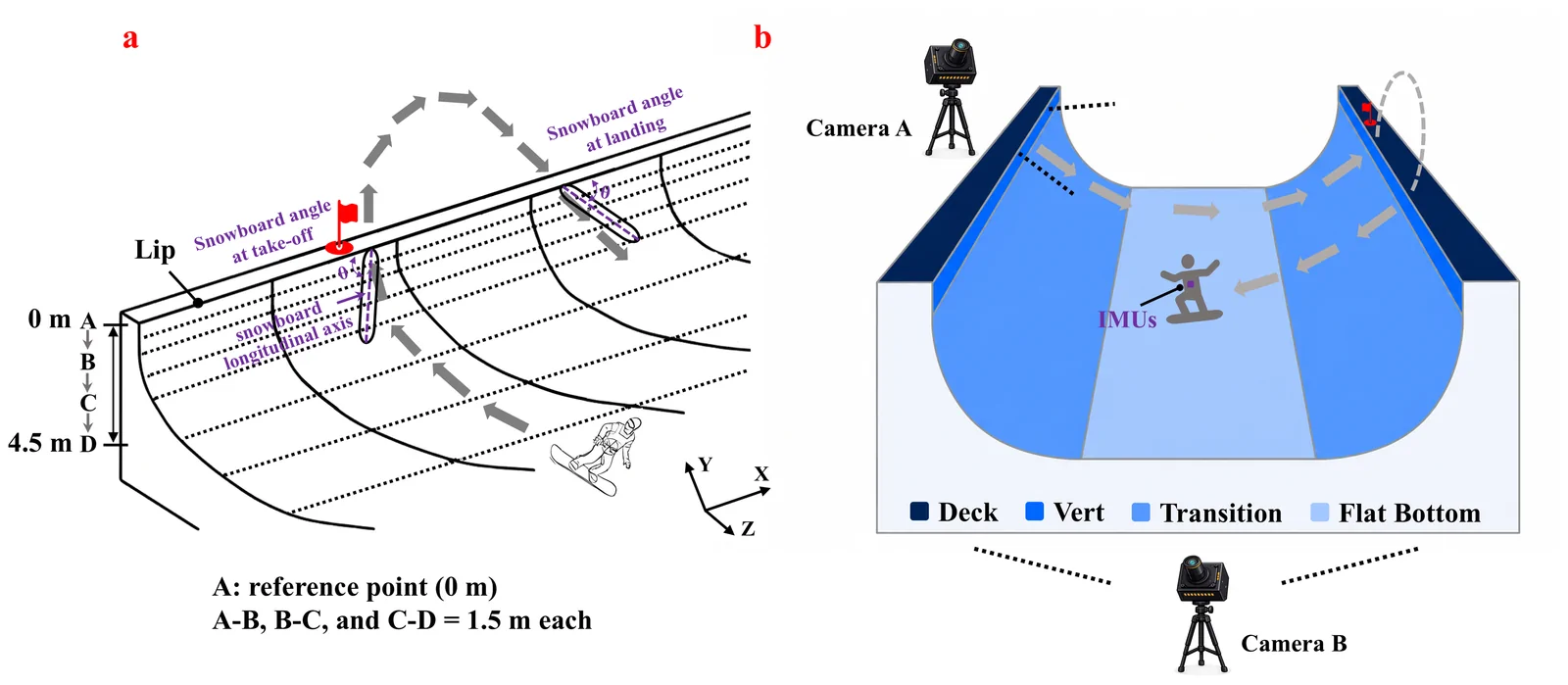
Halfpipe structure, landing-area division, and experimental setup. The halfpipe structure and area classification in B) were redrawn with reference to Merz et al. (2025a).

The landing area of the U-shaped halfpipe was divided into three zones: A–B, B–C, and C–D. Each zone had a vertical height of 1.5 m, and the total vertical height from A to D was 4.5 m (Fig. 2A). The lip (A) was the starting point for calculating the longitudinal distance (Kan et al., 2017).

#### Movement decomposition

The aerial technique was uniformly selected as a frontside 360°. All athletes performed the technique using their natural goofy stance (right foot forward), and switch frontside 360° techniques were not included. We used the structure of the snowboard as a marker to identify the phase of the movement, defining the moment when the athlete completes the first hit and the snowboard fully enters the U-shaped halfpipe as the beginning of the first technical action. The IMU data were first synchronized with the video recordings using the characteristic features of the first take-off as a common event. The first take-off was identified from the video recordings based on the characteristic body posture, and the corresponding movement observed in the Xsens animation was used for temporal synchronization, after which the movement phases were manually labeled. Recent studies have also shown that IMU-based event detection supported by synchronized video labeling is feasible in snowboard halfpipe (Gorges et al., 2024). The athletes performed at least four jumps in one run, each of which was divided into four phases.

In-run: from the moment when the athlete entered the halfpipe and the snowboard completely touched the snow to the moment when the snowboard tip reached the lip. *Take-off*: from the moment when the snowboard’s tip left the lip to the moment when the snowboard’s tail completely left the lip. No clear pre-take-off was identified in the valid trials included in this study. *Flight*: from the moment when the snowboard completely left the halfpipe to the moment when one end of the snowboard touched the snow. *Landing*: from the moment when one end of the snowboard touched the snow to the moment when the snowboard completely touched the snow.

#### Coordinate system and joint angle

Video recordings were first spatially calibrated using a one m reference scale. A two-dimensional coordinate system was then established, with the anteroposterior direction defined as the horizontal axis and the vertical direction defined as the orthogonal axis (Fig. 1A). System calibration of the wearable sensors was performed using a standardized standing–walking protocol (npose + walk), and the acquisition mode was configured at a single level. As this study focused on body kinematics rather than the athletes’ absolute position within the halfpipe, the single level mode was selected to ensure consistency with the study design. Subsequently, a three-dimensional reference frame was constructed, in which the mediolateral direction was defined as the *Z*-axis, while the remaining axes corresponded to those in the two-dimensional setting (Fig. 2A). The origin of the coordinate system for COM calculation was defined based on the athlete’s local coordinate system, with the plantar surface of the foot used as the reference origin. Temporal alignment between the video recordings and IMU signals was achieved by matching predefined key motion events with the corresponding animation timelines generated by the Xsens software. These events were identified from the 500 Hz high-speed video based on the position of the snowboard relative to the halfpipe. They included snowboard entry into the halfpipe, tip contact with the lip, tip leaving the lip, tail leaving the lip, initial snow contact, and full snowboard contact with the snow. The corresponding body movements observed in the Xsens animation were then used for temporal synchronization. After synchronization, the IMU data were labeled according to these events. The matching and labeling procedure was performed manually based on visually identifiable events in the synchronized video and Xsens animation, so that the same predefined criteria were consistently applied across all trials.

The hip joint angle was defined as the angle between the line joining the anterior superior and inferior iliac spines and the long axis of the femur. The knee joint angle was defined as the angle between the extension line of the long axis of the femur and the tibia. The shoulder joint angle was defined as the angle between the humerus and the clavicle. Joint motion directions were coded using a signed convention, in which movements oriented anteriorly, superiorly, and laterally (*e.g.*, flexion, eversion, and abduction) were represented by positive values, whereas movements oriented posteriorly, inferiorly, and medially (*e.g.*, extension, inversion, and adduction) were represented by negative values.

### Participants

In an outdoor experiment, sixteen halfpipe snowboarders from the Chinese Beijing and Hebei provincial teams were divided into elite group (eight halfpipe snowboarders; male; age: 18.00 (17.00–19.00 years); height: 175.00 (172.00–178.00 cm); mass: 57.50 (55.00–60.00 kg); snowboard brand: Burton Custom; years of snowboard halfpipe training: 5.00 (4.00–6.00 years)) and novice group (eight halfpipe snowboarders; male; age: 15.00 (13.00–18.25 years); height: 170.00 (157.00–178.50 cm); mass: 55.00 (40.00–60.25 kg); snowboard brand: Burton Custom; years of snowboard halfpipe training: 3.00 (2.75–3.25 years)). Participant characteristics are presented as median (P25–P75). All athletes in the present study used a goofy stance. The grouping of the athletes was based on an overall assessment of their competitive level in snowboard halfpipe. Specifically, years of snowboard halfpipe training, technical ranking within their respective teams (with the top four athletes in each team assigned to the elite group), and competitive achievements, including selection for the national training team and participation in national-level competitions, were considered when assigning athletes to the elite and novice groups.

All athletes were fully informed of the overall experimental procedures and provided signed informed consent. The study was conducted in accordance with the Declaration of Helsinki, and approved by the Scientific research ethics committee of Shanghai University of Sport (approval number: 102772021RT131; date of approval: 30 December 2021).

### Equipment

The testing equipment included an IMU-based motion capture suit with 17 sensors (240 Hz, Xsens MVN Link, Movella, the Netherlands), which was used in offline mode during data collection and was therefore not limited by transmission distance. Each IMU consisted of a 3D linear accelerometer with a measurement range of ±160 m/s^2^ (±16 g), a 3D rate gyroscope with a measurement range of ±2,000°/s, and a 3D magnetometer. The 17 IMUs were embedded in a Lycra full-body suit and positioned according to the manufacturer’s recommended configuration, with sensors symmetrically placed on the upper limbs, lower limbs, and feet. Specifically, the sensors were placed at the following locations: head (comfortable position on the forehead), sternum (middle of the chest), pelvis (flat on the sacrum), shoulder (scapular region), upper arm (lateral side above the elbow), forearm (lateral side near the wrist), hand (dorsal side of the hand), upper leg (lateral side above the knee), lower leg (flat on the tibia, centered on the medial side), and foot (center of the instep of the snowboard boot). The sensors captured the three-axis attitude angle (or angular rate) and acceleration of each motion segment using built-in sensor fusion algorithms. The Xsens system has been validated in previous studies, showing good agreement with optical motion capture for most joint-angle measurements (Barreto et al., 2021). Two cameras (500 Hz, 2,048 × 2,048 pixels, Fastcam Mini WX100, Photron, Japan), a multifunctional weather station and a snowpack analyser (Snow Fork, Finland), were utilised.

### Procedures

Prior to data acquisition, all recording devices were systematically adjusted and verified, including camera placement, field of view, lens orientation, and exposure settings. Reference markers were then applied to the equipment, and ambient environmental variables were continuously monitored and recorded throughout the experimental session. All data were collected on a single test day. The climatic and environmental parameters on the test day were as follows: the wind speed was 1.3 ± 0.7 m/s; the wind direction was northerly; the temperature was −0.5 ± 2.9 °C; the relative humidity (RH) was 40.9 ± 14.6%; the CO_2_ was 437.3 ± 29.7 ppm; the atmospheric pressure was 90.8 ± 0.1 kPa; in terms of density, the natural snow was 0.3 ± 0.1 g cm^−3^, and artificial snow was 0.5 ± 0.0 g cm^−3^. At the beginning of each testing session, participants’ demographic information and warm-up routines were documented. Athletes were then fitted with the measurement devices and provided sufficient time to become familiar with the equipment. After confirming that the sensors did not cause noticeable discomfort or restrict natural movement, each participant completed three valid runs within a one-hour period. At least four jumps in one run in the U-shaped halfpipe were required to ensure movement quality, and the coach would judge the quality. To minimise the potential effects of physical fatigue, a recovery period of 15–20 min was scheduled between consecutive runs. This interval also matched the practical time required for athletes to return to the start area, prepare for the next run, and maintain a relatively consistent physical state across trials. Environmental conditions remained relatively constant throughout the testing sessions. In addition, camera positioning and lighting were carefully controlled to ensure optimal image quality and consistent recording conditions across all trials. The observation deck built at the bottom of the U-shaped halfpipe trough was converted into a place for athletes to change clothes whilst wearing sensor equipment. A heater was placed inside to maintain the temperature inside the tent. During the recovery period, athletes mainly underwent passive recovery while riding the chairlift back to the start area. They then remained in the heated tent for preparation before the next run, including checking the integrity of the equipment and switching the instruments on and off when necessary to avoid possible IMU drift caused by prolonged activation. Maintaining the tent temperature also helped prevent excessive body cooling and facilitated clothing changes between athletes. All data collection procedures were conducted by trained researchers with expertise in biomechanics, while dedicated technical personnel provided on-site support for sensor placement, trial recording, and system operation.

### Data analysis

Motion data extracted from the recorded videos were processed by trained analysts using Simi Motion 2D/3D (version 7.5.0.285). An experienced biomechanist manually identified the segment centers from the video recordings, and these were connected to construct the athlete-snowboard system. Based on this system, the centre of gravity was calculated, and kinematic variables including snowboard orientation, landing location, and centre-of-gravity velocity were derived. IMU signals were processed offline with Xsens-MVN Analyse (version 2020.0.2) using the Full Body suit configuration, single-level setup, and HD (Slow) processing, and integrated with calibration datasets through the BodyPack platform to generate joint angles and centre-of-mass trajectories. In the present study, the centre of mass was obtained from the IMU-based Xsens MVN system, whereas the centre of gravity was calculated from the video-based Simi Motion 2D/3D system. Therefore, these variables were derived from different measurement systems and were treated as separate variables. Data organisation and preliminary processing were performed in Microsoft Excel (2021), while graphical visualisation was completed using Origin (2024) and Microsoft PowerPoint (2021). The athletes’ take-off and landing data were standardised from the start to the finish of each stage (time normalised), averaged and smoothed using a low-pass filter with a cut-off frequency of eight Hz.

### Statistics

Data are presented as mean ± standard deviation. Due to the small sample size, between-group differences were compared using the Mann–Whitney *U* test, a non-parametric test, with the significance level set at *P* < 0.05.

## Results

### Rhythm distribution

Figure 3 shows the changes in centre of mass position, and the overall pattern of change across the four consecutive movements was broadly similar between the two groups of athletes.

**Figure 3:**
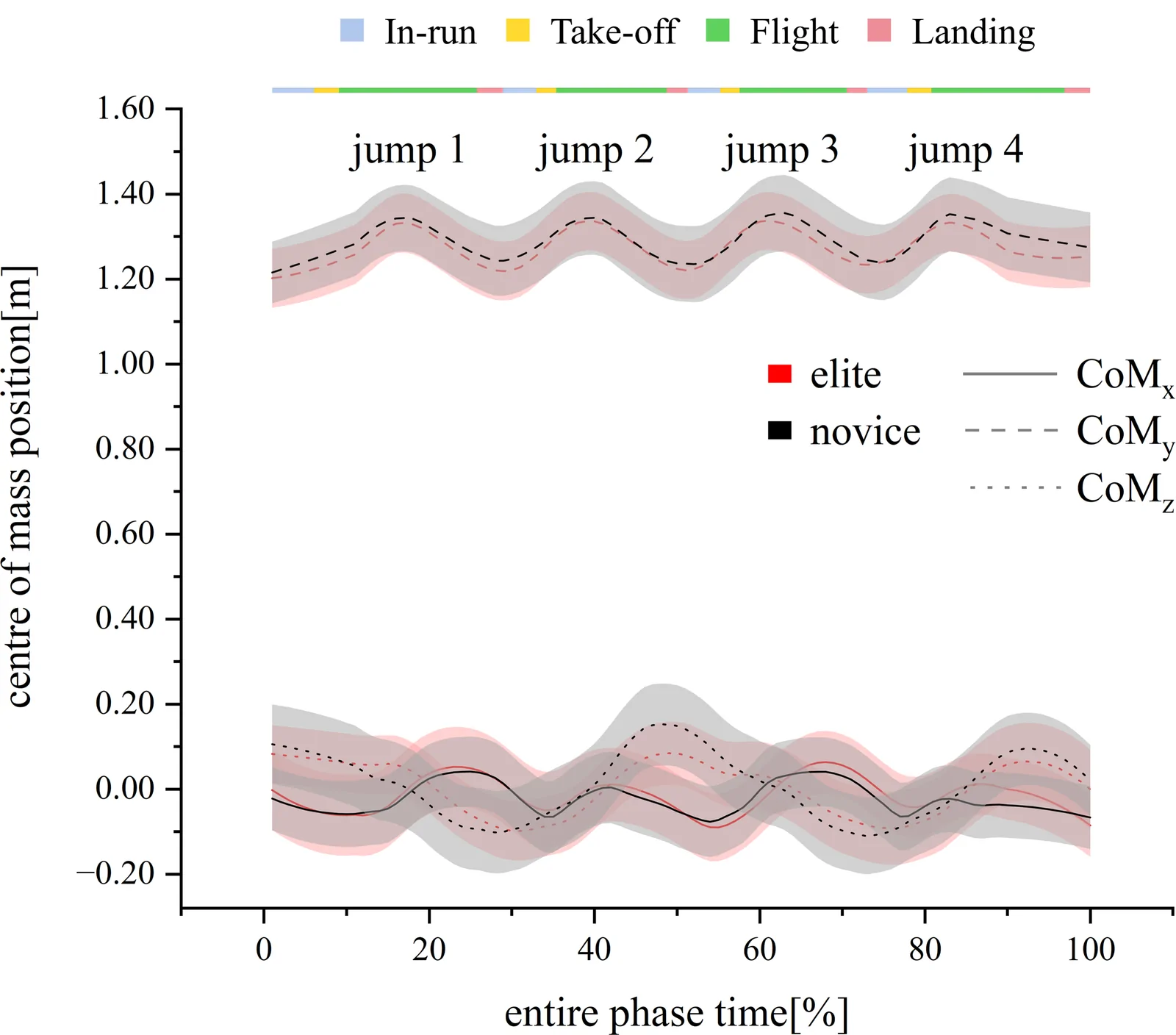
Centre of mass position during one run. Group-averaged centre-of-mass (CoM) position of the athlete–snowboard system during one run, including four consecutive jumps. The CoM position was calculated from IMU data (Xsens, 240 Hz) and expressed in the 3D coordinate system. Solid lines represent the CoM x component in the sagittal direction, dashed lines represent the CoM y component in the vertical direction, and dotted lines represent the CoM z component in the frontal direction. Shaded areas represent standard deviations. Red and black curves indicate the elite and novice groups, respectively. The colored bars above each jump indicate the average relative durations of the movement phases (in-run, take-off, flight, and landing) within the normalized jump cycle.

Table 1 presents the temporal proportions and durations of each phase, and the small differences in the mean time spent on each jump (1–4) between the two groups suggest relatively stable time control during performance.

**Table 1:** Comparison of phase durations between the elite and novice groups.

**Group**	**In-run (s)**	**Take-off (s)**	**Flight (s)**	**Landing (s)**	**Total duration (s)**
Elite	1.202 ± 0.080	0.127 ± 0.033	0.522 ± 0.106	0.110 ± 0.025	1.962 ± 0.092
Novice	1.201 ± 0.048	0.132 ± 0.036	0.512 ± 0.079	0.117 ± 0.023	1.961 ± 0.062
*P*-value	0.976	0.776	0.834	0.569	0.980

### Take-off

Figure 4A shows that the resultant velocity of the athlete’s centre of gravity decreased as the upward slope of the wall increased in the take-off phase. The X velocity component of all athletes gradually increased, whilst the Y velocity component continued to decrease. Figure 4B shows that the flexion angles of the left and right hip initially increased slightly and then gradually decreased. The amplitude of change was relatively small, and the change patterns on both sides were very similar. Figures 4C and 4D show that the changes in the left and right knee joints varied. The knee flexion angle gradually decreased during the first 70% of the take-off phase, with the left knee (rear leg) more drastically changing and rapidly decreasing, whilst the right (front leg) knee flexion angle more gradually changed. The left knee flexion angle gradually increased during 30% of the take-off phase, whilst the right side continued to decrease. Figures 4E and 4F show that at the start of the take-off phase, the right arm is flexed in front of the body, and the left arm is extended behind the body. Subsequently, the left arm is moved forward and bent in front of the body, whilst the right arm is gradually extended behind the body, forming a swinging motion that caused the torso to twist.

Table 2 shows that the take-off angle of the snowboard for elite athletes was 56°, the change value after the take-off was about 9°, the take-off angle of the snowboard for novice athletes was 57°, and the change value was about 16°.

**Figure 4:**
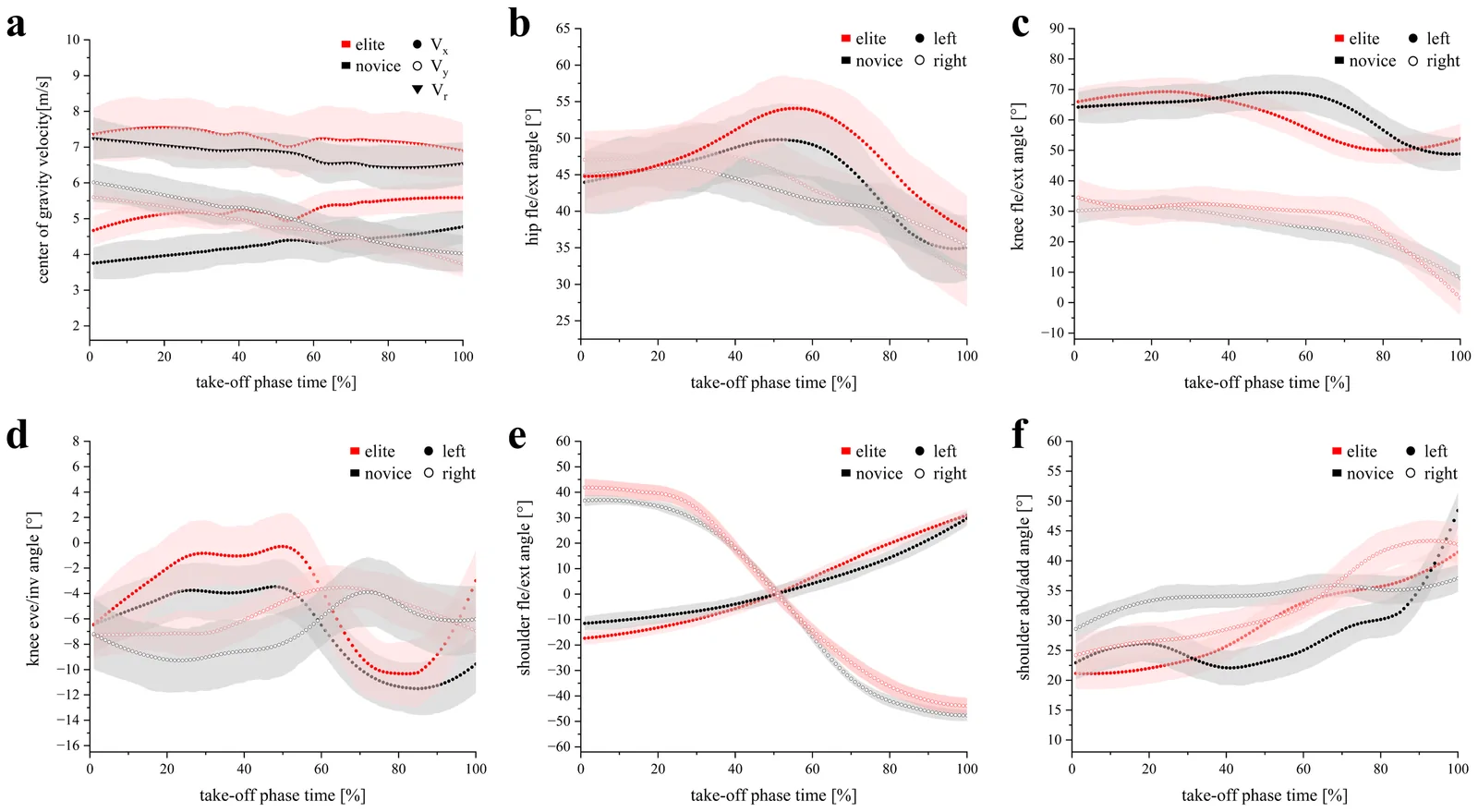
Take-off phase kinematic parameters. Group-averaged kinematic parameters during the take-off phase for the elite and novice groups. (A) Centre-of-gravity velocity parameters calculated from 2D video data (Fastcam Mini WX100, 500 Hz), including the X velocity component (Vx), Y velocity component (Vy), and resultant velocity (Vr). (B–F) Joint angle parameters calculated from IMU data (Xsens, 240 Hz), including key angles of the hip, knee, and shoulder joints. Red and black curves indicate the elite and novice groups, respectively. In panel (A), filled circles represent Vx, open circles represent Vy, and filled triangles represent Vr. In panels (B–F), filled circles represent the left side and open circles represent the right side. Shaded areas represent standard deviations. flex/ext = flexion/extension; eve/inv = eversion/inversion; abd/add = abduction/adduction.

Table 3 presents the take-off phase technical parameters of the two groups. The elite group showed higher maximum and minimum Vx velocities and greater ROM in left shoulder flexion/extension, right shoulder abduction/adduction, and right knee flexion/extension, whereas the novice group showed greater left shoulder abduction/adduction ROM.

### Landing

Figure 5A shows that the resultant velocity of the athlete’s centre of gravity increased with the decrease in the height in the landing phase. The X and Y velocity component of all athletes gradually increased. The hip and knee flexion angles of both groups of athletes during the landing phase demonstrated a discernible pattern. Specifically, a decrease followed by an increase is observed in the first 30% of the action phase and then a decrease in the subsequent 70% (Figs. 5B, 5C and 5D). The angle of the shoulder indicated that the motion of the upper limb is split on both sides and did not make contact with the ground (Figs. 5E and 5F).

**Table 2:** Comparison of snowboard angles during the take-off phase. The angle was calculated from 2D video data (Fastcam Mini WX100, 500 Hz) and defined as the angle between the snowboard longitudinal axis and the lip reference line at the start and end of the take-off phase.

**Group**	**Snowboard angle at take-off start (^∘^)**	**Snowboard angle at take-off end (^∘^)**
Elite	55.64 ± 3.63	46.50 ± 2.48
Novice	57.28 ± 5.58	41.69 ± 4.47
*P*-value	0.500	0.023

**Table 3:** Comparison of take-off phase technical parameters between the elite and novice groups. Vx and Vy represent velocity components, and Vr represents resultant velocity. ROM, range of motion, was calculated as the maximum value minus the minimum value.

**Parameters**	**Elite**	**Novice**	*P*-value
Maximum Vx velocity (m/s)	5.59 ± 0.44	4.77 ± 0.46	0.003
Maximum Vy velocity (m/s)	5.60 ± 0.36	6.01 ± 0.54	0.099
Maximum Vr velocity (m/s)	7.56 ± 0.80	7.24 ± 0.59	0.379
Minimum Vx velocity (m/s)	4.67 ± 0.41	3.76 ± 0.44	<0.001
Minimum Vy velocity (m/s)	3.73 ± 0.36	4.03 ± 0.50	0.189
Minimum Vr velocity (m/s)	6.91 ± 0.77	6.43 ± 0.63	0.196
Left shoulder flex/ext ROM (^∘^)	48.32 ± 3.72	41.26 ± 3.95	0.002
Right shoulder flex/ext ROM (^∘^)	84.67 ± 2.94	85.76 ± 4.59	0.587
Left shoulder abd/add ROM (^∘^)	20.33 ± 3.85	26.33 ± 4.14	0.011
Right shoulder abd/add ROM (^∘^)	19.15 ± 4.87	8.47 ± 3.23	<0.001
Left hip fle/ext ROM (^∘^)	16.72 ± 6.40	14.93 ± 6.81	0.599
Right hip fle/ext ROM (^∘^)	16.98 ± 5.86	12.80 ± 5.32	0.161
Left knee fle/ext ROM (^∘^)	19.30 ± 6.27	20.29 ± 8.06	0.791
Right knee fle/ext ROM (^∘^)	32.95 ± 8.37	23.39 ± 5.32	0.023
Left knee eve/inv ROM (^∘^)	10.04 ± 3.64	7.76 ± 2.98	0.190
Right knee eve/inv ROM (^∘^)	3.75 ± 2.52	5.39 ± 3.73	0.344

**Figure 5:**
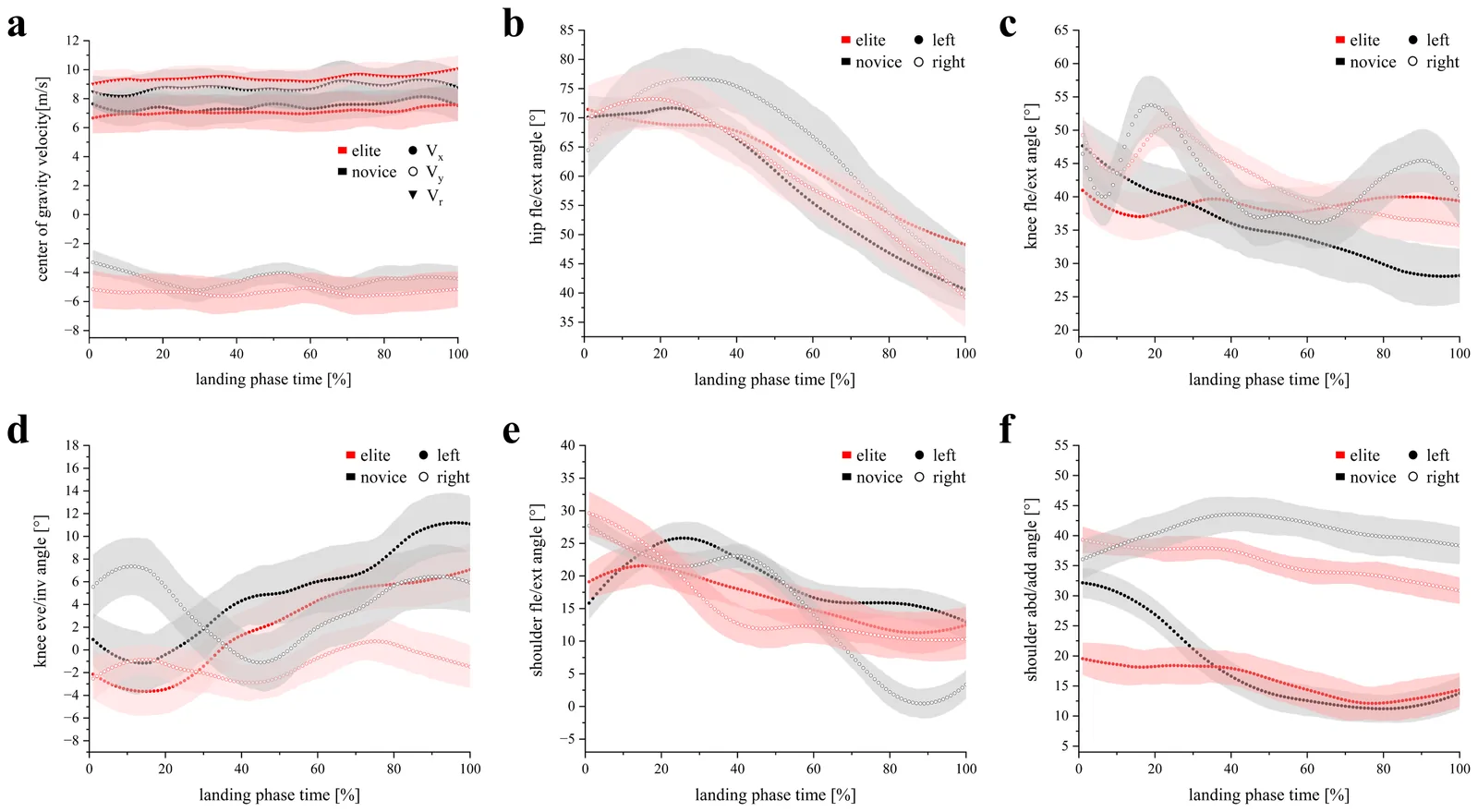
Landing phase kinematic parameters. Group-averaged centre-of-gravity velocity and joint angle parameters during the landing phase for the elite and novice groups. The variables, symbols, line types, colours, and shaded areas are defined as in Fig. 4.

Table 4 shows that the landing angle of the snowboard was 30° for elite athletes and 34° for novice athletes. The landing position of novice athletes was 0.68 ± 0.08 m, located in the A–B region, whereas that of elite athletes was 1.94 ±0.12 m, located in the B–C region.

**Table 4:** Comparison of snowboard angle and landing position between the elite and novice groups. The snowboard angle was calculated from 2D video data (Fastcam Mini WX100, 500 Hz) and defined as the angle between the snowboard longitudinal axis and the horizontal reference line parallel to the lip at the moment of full landing. The landing position was calculated from the lip (point A) downward along the halfpipe wall to the location where the snowboard completely touched the vertical surface or transition area (Fig. 2A).

**Group**	**Snowboard angle at landing (^∘^)**	**Landing position (m)**
Elite	30.12 ± 2.68	1.94 ± 0.12, zone B-C
Novice	34.00 ± 7.14	0.68 ± 0.08, zone A-B
*P*-value	0.219	<0.001

Table 5 presents the landing phase technical parameters of the two groups. The elite group showed a larger magnitude of minimum Vy velocity in the negative direction, whereas the novice group showed greater ROM in right shoulder flexion/extension, left shoulder abduction/adduction, left hip flexion/extension, left knee flexion/extension, and right knee eversion/inversion.

**Table 5:** Comparison of landing phase technical parameters between the elite and novice groups. Negative Vy values indicate the direction of the vertical velocity component.

**Parameters**	**Elite**	**Novice**	*P*-value
Maximum Vx velocity (m/s)	7.58 ± 1.15	8.15 ± 1.03	0.314
Maximum Vy velocity (m/s)	−5.64 ± 1.28	−5.22 ± 0.83	0.451
Maximum Vr velocity (m/s)	10.08 ± 0.91	9.31 ± 1.32	0.198
Minimum Vx velocity (m/s)	6.67 ± 1.06	7.08 ± 1.03	0.446
Minimum Vy velocity (m/s)	−5.06 ± 1.06	−3.30 ± 0.87	0.003
Minimum Vr velocity (m/s)	9.03 ± 0.91	8.18 ± 1.33	0.161
Left shoulder flex/ext ROM (^∘^)	10.29 ± 4.27	12.86 ± 3.68	0.219
Right shoulder flex/ext ROM (^∘^)	19.45 ± 4.64	27.26 ± 3.18	0.002
Left shoulder abd/add ROM (^∘^)	7.43 ± 3.83	20.95 ± 3.39	<0.001
Right shoulder abd/add ROM (^∘^)	8.47 ± 3.11	7.50 ± 4.35	0.617
Left hip fle/ext ROM (^∘^)	23.14 ± 6.21	31.07 ± 5.20	0.015
Right hip fle/ext ROM (^∘^)	33.95 ± 7.36	33.12 ± 7.06	0.821
Left knee fle/ext ROM (^∘^)	3.97 ± 5.04	19.58 ± 5.81	<0.001
Right knee fle/ext ROM (^∘^)	14.96 ± 4.39	17.71 ± 5.53	0.290
Left knee eve/inv ROM (^∘^)	10.71 ± 3.18	12.36 ± 3.72	0.357
Right knee eve/inv ROM (^∘^)	3.64 ± 2.41	8.46 ± 3.56	0.008

## Discussion

The main findings of this study were that athletes of different performance levels showed distinct technical characteristics during the take-off and landing phases of snowboard halfpipe. In general, the results supported our hypothesis that elite athletes would demonstrate more stable and better-controlled take-off and landing movements than novice athletes. Specifically, the elite athletes tended to show earlier take-off timing, better snowboard control, and a landing position further down the halfpipe transition. These findings provide a basis for further discussion of the technical characteristics associated with take-off quality and landing stability.

This study described the group-averaged rhythm distribution across four consecutive jumps within one run. The body position showed relatively small fluctuations in the anterior-posterior and left–right directions, indicating stable sliding posture control (Fig. 3 and Table 1). The centre of mass gradually increased during the take-off phase and decreased during the landing phase. Only minor differences were observed between the two groups. The duration of the take-off and landing movements was shorter than that of the flight and in-run. The former was a preparatory stage for flight movements, whilst the latter was a transitional process for the next jump and should not be allowed to make irreparable mistakes. These two phases placed high demands on the proficiency and timing of the athletes’ technical movements and should be given special attention by researchers and coaches.

### Take-off

Athletes must mobilise their entire body muscles in a short amount of time and work together to apply force to the groove wall through the snowboard in a rapid and coordinated manner. A short and rapid increase occurred as the velocity drops to its lowest point (Fig. 4A), as the athlete performed a take-off from the vertical to gain reaction force by extending the knees and hip joints, thereby accumulating kinetic energy (Li & Cheng, 2018). The progressive action mode of the lower limbs during take-off possibly has a greater effect on the X velocity component in the take-off phase (Platzer et al., 2009). The overall speeds achieved by the elite athletes were greater than those of the novice athletes (Table 3).

The lower limb angles showed a regular change during the take-off phase (Figs. 4B, 4C and 4D). This phenomenon was due to the left lower leg causing the snowboard to bend towards the thigh in preparation for the spin after take-off. The analysis of the lower limb angle results showed that elite athletes exhibited greater activation and extension in their lower limbs compared with novice athletes. The knee joint flexion angle decreased faster than that of the hip joint flexion angle after take-off. Therefore, the action mode of two groups of athletes in the take-off movements was driven by the ‘knee joint driving the hip joint’.

This study did not show any drastic changes in the hip and knee angles of the lower limbs in flexion and extension in either of the group of athletes. American snowboard coach Logan (Brousseau & Desbiens, 2018) believed that, unlike jumping on a flat ground or on a slope with a small angle, the snowboarders should gradually push their body away from the inner wall along the transition zone when taking off in a U-shaped area. A sudden explosive movement of the lower limbs should be avoided. The goal should be a natural transition, gradually increasing the position of the body’s centre of mass in relation to the snowboard. The snowboard gradually transitioned down the path, slid out along the vertical and took off relying on inertia. The experimental results of this study further confirmed the above-mentioned idea. This softer and inertia-enabled take-off technique ensured a minimal shift in the take-off angle, thus increasing take-off heights and improving parabolic trajectories.

The elite athletes reached the vertical position at approximately 50% of the normalized take-off phase, compared with approximately 60% in novice athletes. During take-off from the vertical position, the snowboard was generally observed to extend beyond the deck by less than one-third of its longitudinal axis. Therefore, the tip of the snowboard was used as a positional reference point. When the difficulty level of the air was low, the excess portion of the snowboard should be reduced, whilst it should be increased when the difficulty level is high. The take-off action also had a significant effect on the take-off angle of the snowboard. The change value of the snowboard after the take-off for elite athletes was about 9°, and that for novice athletes was approximately 16° (Table 2). The above-mentioned alteration in angle showed that the take-off action altered the take-off inclination of the snowboard. Furthermore, the adjustments in shoulder joint angle revealed that the athlete’s torso exhibited a pre-swing rotation motion in the initial take-off phase, driving the lower limb rotation and affecting the take-off angle of the snowboard. Li, Li & Zhou, (2014) research revealed a deficiency in the take-off angle of low-level male snowboard halfpipe athletes. This result was consistent with the findings of this study. When the take-off angle was inadequate, athletes struggled to convert their horizontal velocity into vertical velocity, resulting in an inability to gain height in flight and execute challenging aerial movements. Recent biomechanical modelling further suggested that, although both airtime and angular velocity contribute to rotational performance, angular velocity has a stronger influence than airtime (Merz et al., 2026). Identification of the appropriate angle in advance was important to ensure that the proper take-off angle of the snowboard was achieved whilst maintaining a specific take-off velocity (Chen, 2015).

The change in the left and right arm positions, forming a swinging motion that caused the torso to twist (Figs. 4E and 4F), indicate a synergistic relationship between the arms and the torso, where arm movements drive the torso. The abduction angle of the shoulder joint gradually rose, which elevated the centre of gravity and lengthened the moment arm of the body during take-off, resulting in a higher initial torque (Mkaouer et al., 2013). Yan, Liu & Guo (2009) pointed out that abducting the arms during the take-off phase could increase the moment of inertia, resulting in a greater rotational angular velocity during take-off and flight. The shoulder angle indicated only a minor difference in the posture angle of the torso twist between the two groups of athletes. Related evidence has also been reported in snowboard big air, in which the take-off phase was identified as critical for subsequent flight trajectory, rotational performance, and the execution of higher-difficulty maneuvers (Jiang et al., 2025). The above-mentioned purposeful ‘separation’ between the arms and torso and the lower limbs prior to take-off produced a rotational motion in the opposite direction of the flipping action in air. This motion could additionally transmit angular momentum and result in a synchronised rotation of the snowboarder and snowboard. However, when the torso and arms finished the ‘preparation action’, they should pay more attention to controlling the relative position of the centre of gravity with the snowboard; otherwise, changes in body mass distribution could affect the rotational inertia of the human body, resulting in early rotation of the snowboard or increased force on one edge of the snowboard caused by a shift in the centre of gravity position. This situation can affect flight height and stability (Su, 2020; King, 2005). Thus, preparatory movements must be initiated as early as possible when performing complex aerial movements with a high number of flips, and *vice versa*.

### Landing

The athlete’s lower limb range of motion, the hardness of the snow, the slope of the landing surface and the direction of movement of the snowboard contributed to the reduction in the impact force of the snow surface on the snowboard in the landing phase (O’Shea, 2004). Therefore, the change in landing body posture, the landing angle of the snowboard and the landing position were critical factors influencing the stability of the landing phase.

These findings of lower limb angles suggested that the lower limbs of athletes maintained a gradual extension posture even when one end of the snowboard touched the snow surface upon completing the aerial movement (Figs. 5B, 5C and 5D). The impact on the athlete’s lower limbs increases as the contact area between the snowboard and snow surface gradually increased. Consequently, the hip and knee joints of the athlete quickly flex to cushion the landing and lower the centre of gravity for stability. Subsequently, the lower limbs gradually extended to raise the centre of gravity. The result of the comparison of the hip and knee joints demonstrated that the leg making the initial contact with the snow surface during the landing phase rapidly flexed in a knee-driven hip pattern. Meanwhile, the other leg that made a delayed contact exhibited a similar pattern of flexion but with a smaller range. When the body was stabilised, the hip and knee joints rapidly extended in a hip-driven knee pattern, with the hip extending faster on the front leg in the direction of the landing.

Landing strategies were typically classified as either ‘soft landing’ or ‘hard landing’, with a soft landing being defined as one in which the range of motion of the human knee joint in flexion exceeded 63° and a hard landing being one in which it was less than 63° (Assenmacher & Hunter, 2002). Soft landings could effectively lessen the impact of the ground reaction forces on the lower limbs whilst decreasing the odds of knee injury. Nevertheless, lower limb joint mobility during soft landings resulted in increased forward shifting of the body’s centre of gravity, lowering stability (Guy-cherry et al., 2018). However, hard landings increased tibia forward shift, heightening knee injury risk but providing higher stability during landing (Markolf et al., 2019). The alteration in knee flexion angle evidently showed that both groups of athletes exhibited knee flexion activity angle of less than 63° in the landing phase. Both groups also implemented a hard landing tactic to enhance stability. It should also be noted that the classification and interpretation of landing strategy in snowboard halfpipe may be influenced by the landing location, because ground reaction forces have been reported to be higher in the transition than in the vertical section of the halfpipe (Thelen et al., 2025). The elite group demonstrated a significantly reduced flexion range of motion and a stiffer landing in contrast to the novice group (Table 5). The velocity of the centre of gravity was represented by the trend of the Y velocity component of the novice group athletes during the landing phase. This trend showed an initial increase, followed by a decrease and then a gradual increase (Fig. 5A). The change in velocity indicated that the snowboard and the human body actively absorbed part of the landing impact, which corresponded to the angular result of the buffering movement. Furthermore, the combination of video images and changes in the shoulder angles indicated that the upper limbs were divided on both sides, spread forward and backward and mainly play a role in maintaining balance when landing, without any contact with the ground (Figs. 5E and 5F). This motion acted to uphold equilibrium and heighten stability (Hébert-losier, Supej & Holmberg, 2014; Pontzer et al., 2009). The inclination towards the choice of landing approach did not preclude the possibility that the aerial movements used in the experiment had a low complexity coefficient, and further research was required.

In practice, snowboarders found it challenging to maintain a consistent angle of inclination between the snowboard and the inner wall during the halfpipe phase. When the snowboarder entered the transition area, one end of the snowboard made contact with the snow surface before the rest of the snowboard gradually followed. This sequence of events posed an elevated risk of lower limb injury to the athlete on the side where they landed first (Ouyang & Chen, 2023). The landing angle for the snowboard was 30° for elite athletes and 34° for novice athletes (Table 4). The landing snowboard angle may be related to the in-air adjustment of snowboard orientation before touchdown. During the flight phase, athletes needed to adjust the snowboard angle in preparation for landing. Consequently, the extent of this adjustment may have affected the final landing angle of the snowboard. The difference in angle indicated that the two groups showed different snowboard orientation characteristics at landing when completing the same 360° movement.

Kan et al. (2017) conducted a statistical analysis of where elite and novice athletes landed in the snowboard halfpipe. The results showed that elite athletes prefer to land in the B–C and C–D areas, whilst novice athletes typically concentrate on the A–B and B–C areas. The A–B area was problematic because it was considerably close to the lip, potentially causing the snowboard to hit the wall and the head to bump, resulting in a fall, more serious injuries and even failure of the set sequence of movements. Analysis of the video images showed that novice athletes landed in the A–B region (0.68 ± 0.08 m), whilst elite athletes landed in the B–C region (1.94 ±  0.12 m) (Table 4). This difference may reflect different landing strategies between performance levels, with novice athletes tending to adopt a more conservative landing position closer to the lip to facilitate landing control, whereas elite athletes tended to adopt a landing position further down the halfpipe transition to better maintain speed and continuity into the subsequent transition. The angle between the direction of sliding and the curve tangent line of said wall would vary as the athlete entered the transition area from different positions due to the curved design of the inner wall of the U-shaped halfpipe. The proximity of the landing position to the upper section of the inner wall of the U-shaped halfpipe decreases the normal force of gravity, whereas proximity to the lower section increases it (Su, 2020). Consequently, selecting a landing position that was considerably high may lead to the snowboard hitting the lip or vertical. Meanwhile, a landing position that was significantly low can result in a higher impact force on the athlete when entering the transition area. The elevated landing position may also stem from the low complexity of the aerial movements utilised in this experiment. When aerial techniques were elementary and easy to regulate, athletes opted for a higher A–B landing area, thus guaranteeing superior landing quality, whilst also securing greater acceleration into the U-shaped halfpipe, affording more time for glide direction adjustments. When attempting difficult movements, landing at a relatively low position within the B–C area was advisable to prevent excessive impact, which could result in loss of control of the body’s centre of gravity. This approach also allowed for a relatively high speed when entering the flat bottom.

This study had some limitations. Firstly, the sample size was small. Although non-parametric tests were added in the revised analysis, the findings should still be interpreted with caution and are mainly applicable to the present experimental sample. Accordingly, further tests with larger samples were required. Secondly, the technical difficulty set for the athletes was relatively low, resulting in a consistent technique throughout the entire continuous state, making it an ideal training mode. Furthermore, this study considered the safety of the athletes and the acceptance of new equipment. In reality, athletes faced significant challenges in implementing various types of movements. Thirdly, the data were time-normalized within each movement phase rather than location-normalized, which may not fully account for differences in spatial position between athletes moving at different speeds. Future studies should consider location-normalized analysis, include larger samples and more difficult movements, and be conducted closer to competition conditions to compare movement patterns more precisely at equivalent positions within the halfpipe.

From a practical perspective, the findings of this study suggest that technical training in snowboard halfpipe should focus on the control of the transition into take-off and the regulation of landing posture. In particular, coaches and athletes should place greater emphasis on take-off timing, snowboard inclination control, and lower-limb coordination during landing, as these factors may influence movement stability and overall technical performance. In addition, the present study suggests that the combined use of IMU-based motion capture and high-speed video analysis is feasible in a real halfpipe environment, thereby supporting field-based technical evaluation and training feedback.

## Conclusions

This study explored the application of an IMU-based motion capture system combined with high-speed video recording in snowboard halfpipe to quantify key technical characteristics of the take-off and landing phases and to identify technical differences associated with performance level. The findings suggest that snowboard halfpipe performance may depend on the coordinated control of take-off timing, snowboard inclination, and landing posture. Descriptively, the elite athletes tended to show earlier take-off timing, better snowboard control, and a landing position further down the halfpipe transition than the novice athletes. During landing, rapid flexion of the hip and knee joints appeared to be important for impact absorption and stability maintenance. These findings support the potential application of wearable IMU-based motion capture systems in routine training for technique evaluation and technical feedback in snowboard halfpipe.

## Additional Information and Declarations

### Data Availability

The following information was supplied regarding data availability: The data is available at Figshare: Yu, Jinglun (2026). Raw data (Supplementary File 3). figshare. Dataset. https://doi.org/10.6084/m9.figshare.31240120.v1.
